# London Child Labour

**Published:** 1920-02-14

**Authors:** 


### London Child Labour.

New regulations have been adopted by the London County Council governing the employment of children. There was some considerable opposition to allowing children to work at all, but the regulations were passed, the main provisions being as follows:

No child under fourteen to be employed without examination by the school medical officer and without notice having been given to the headmaster.

Hours of such employment to be limited to one hour before school-time and one hour after school-time, the former period being restricted to the sale or delivery of newspapers or to light household work.

No child under these regulations to be employed for more than three hours on a Saturday or for more than five hours on weekdays (excluding Saturdays) when the school is not open.

In inclement weather children working out of doors to be provided with waterproof coat or similar garment.

No Sunday work is to be permitted.

It was urged by several speakers who opposed child labour that the country as a whole was waiting to see what lead London would give in the matter. A few progressive towns like Ipswich and Blackpool had already abolished school-child labour, and if London took the same step they were sure that many other places would follow the example.

The strongest arguments used by the opponents of the employment of school children were the medical and the educational. Medical officers of health, it was declared, had reported unfavourably with a remarkable unanimity of opinion, and those of Devonshire, Plymouth, Gloucestershire, Hertford, and Lancashire were quoted.

The regulations appear to allow considerable chances of abuse, even if they are rigidly enforced, and to permit a child under fourteen to work five hours daily during its holidays can hardly appeal to those who believe that young children should have as much of the brightness and irresponsible happiness of childhood as can be made theirs. Doubtless many parents find it useful for their children to add to the family exchequer, and some, perhaps, need such help. But the latter case ought to be remedied by other means, not at the expense of the children, and no quarter should be allowed in the former case. Public bodies, also, should exercise great care that no encouragement is given to those who exploit child labour because it is cheap. That is a sort of cheapness which costs dear in several important respects, not the least of which is the interest of the child itself.

